# Development of Seborrheic Keratosis and Human Papillomavirus (HPV) Infection Following Four Months of Fingolimod Treatment in a Patient With Multiple Sclerosis: A Report of a Rare Case

**DOI:** 10.7759/cureus.83652

**Published:** 2025-05-07

**Authors:** Giorgi Mamardashvili, Nazibrola Botchorishvili, Gucha Kobaidze, Marina Janelidze

**Affiliations:** 1 Neurology, Tbilisi State Medical University, Tbilisi, GEO; 2 Neurology, Simon Khechinashvili University Hospital, Tbilisi, GEO

**Keywords:** disease-modifying therapy, fingolimod, hpv infection, relapsing-remitting multiple sclerosis, seborrheic keratosis

## Abstract

Multiple sclerosis (MS) is the most common demyelinating disease of the central nervous system in young adults. Disease-modifying therapies (DMTs) for MS target different aspects of the immune system and have various safety profiles. Fingolimod is a DMT introduced for the treatment of MS. Cutaneous adverse events have been well described in patients treated with fingolimod. We present a clinical case of a patient with relapsing-remitting multiple sclerosis (RRMS) who developed multiple skin lesions after four months of treatment with fingolimod. Diagnosis of seborrheic keratosis, nevi, and papilloma was made clinically based on characteristic lesion morphology. Histologic confirmation was not performed, and human papillomavirus (HPV) DNA testing was positive. Rapid progression of cutaneous lesions prompted the switch to a different DMT, after which the lesions resolved completely within two months.

## Introduction

Multiple sclerosis (MS) is a chronic inflammatory autoimmune demyelinating disease of the central nervous system (CNS). There are four clinical forms of MS: relapsing-remitting MS (RRMS), secondary progressive MS (SPMS), primary progressive MS (PPMS), and progressive relapsing MS (PPRMS) [1].

More than half of the patients present with RRMS, which is characterized by acute attacks (relapses) followed by partial or full recovery (remission) [2]. Disease-modifying therapies (DMTs) help to control the disease progression by reducing immune-mediated inflammation [1].

Fingolimod, a sphingosine-1-phosphate receptor (S1PR) modulator, is an oral DMT approved for RRMS. Although generally well-tolerated, it is associated with a variety of adverse events (AEs) that should be considered before and during treatment [3]. Fingolimod-related AEs may affect multiple organ systems, including the hepatic, cardiovascular, and hematologic systems, and are associated with increased susceptibility to infections such as human papillomavirus (HPV) and herpes virus infections [4,5].

Dermatologic manifestations reported in association with fingolimod include skin and mucosal warts, rosacea, and various forms of skin cancers, including squamous cell carcinoma, basal cell carcinoma, and melanoma [4,5]. The onset of these cutaneous AEs varies, ranging from two months to 12 years after treatment initiation [4,6,7]. Therefore, it is recommended that patients undergo a baseline dermatologic assessment and receive annual skin examinations thereafter [8]. Many of fingolimod’s AEs are believed to result from its pharmacodynamic actions on S1PRs, which modulate immune cell trafficking and other systemic responses [8].

This case emphasizes a rare presentation of dermatologic complications in a patient receiving fingolimod therapy for RRMS and underlines the importance of regular dermatologic monitoring. While various cutaneous AEs have been reported, the abrupt onset of multiple seborrheic keratoses in this context is exceedingly uncommon and, to our knowledge, has been scarcely described in the literature.

## Case presentation

A 36-year-old female with a four-year history of RRMS presented with new-onset cutaneous lesions. She had been taking 0.5 mg of fingolimod daily for the past four months. Approximately three to four weeks before admission, she had noticed unusual genital papules and an increased number of “black dots” on her neck and chest, which she described as mildly itchy but non-painful.

Past medical history was significant only for RRMS. There were no known drug allergies, and the patient was not on any immunosuppressant or corticosteroids except for fingolimod.

**Figure 1:**
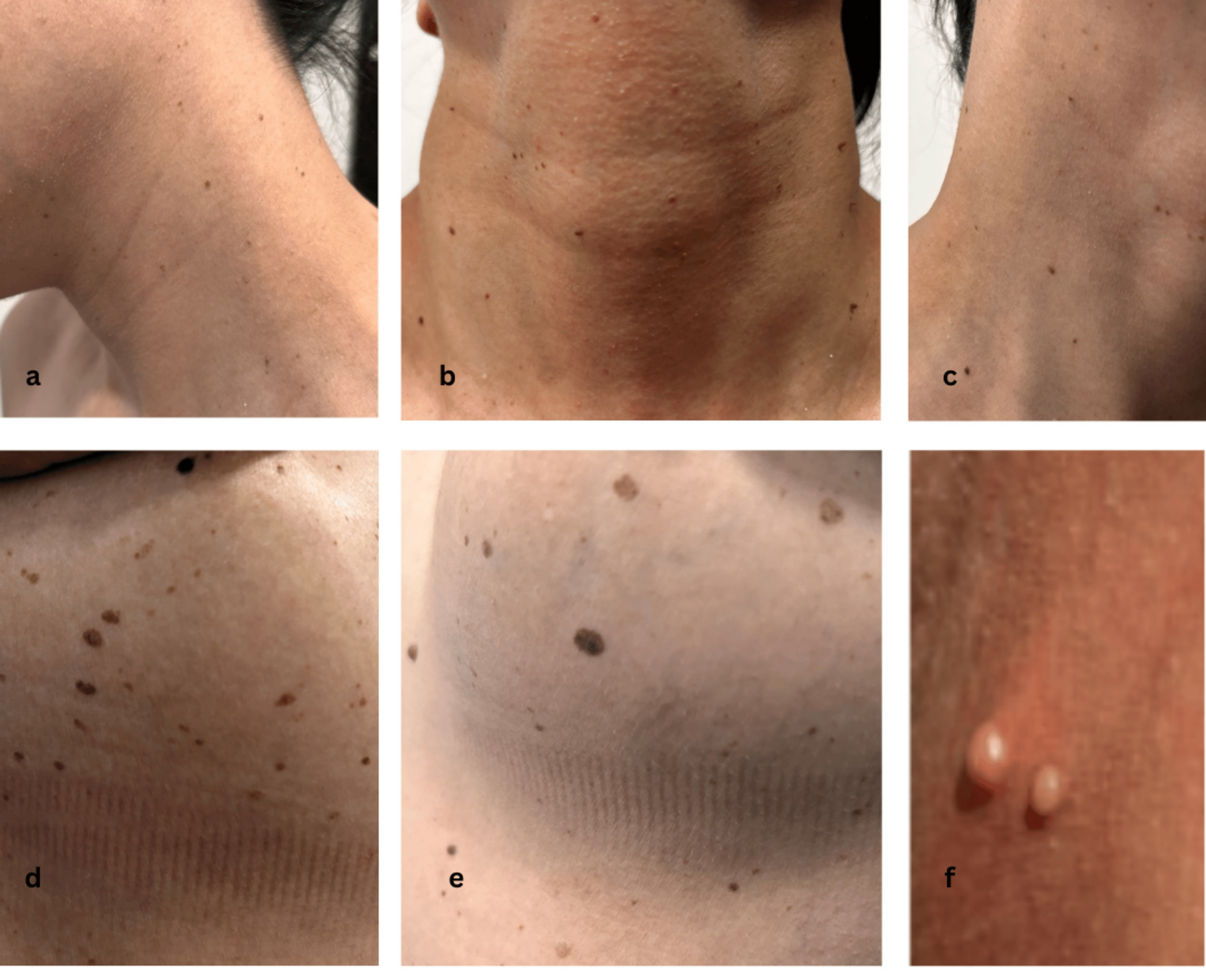
(a-c) Multiple papillomas and seborrheic keratoses on the neck; (d, e) seborrheic keratoses and nevi on the chest and abdomen; (f) genital warts.

Physical examination was significant for multiple hyperpigmented papules and plaques on the neck, chest, and upper abdomen, consistent with seborrheic keratosis and nevi (Figures 1a-1e). In the genital area, several small papillary lesions were observed, raising concern for viral infection (Figure 1f). The lymph nodes were not palpable, and the rest of the physical examination was normal, including neurological assessment.

A complete blood count (CBC) was significant for decreased B lymphocytes. Her white blood cell count and T lymphocyte count were within the normal range (Table 1).

**Table 1:** Summary of relevant laboratory findings Key immunologic parameters at the time of presentation. B-cell lymphopenia was observed, a known effect of fingolimod, which may have contributed to the development of cutaneous human papillomavirus (HPV)-related lesions.

Parameter	Result (at admission)	Reference range	Interpretation
B lymphocytes (CD3⁻ CD19⁺)	4.30%	7.2-22.5%	Decreased
B lymphocytes (CD3⁻ CD19⁺)	41 cells/µL	100.0-600.0 cells/µL	Decreased
T lymphocyte count (CD3⁺)	70%	63.9-72.3%	Normal
T lymphocyte count (CD3⁺)	656 cells/µL	404.0-2075 cells/µL	Normal
Total white blood cell count	6.17 ×10^3^/µL	4.0-10.0 ×10^3^/µL	Normal

Cutaneous cytology on the patient’s skin lesions did not demonstrate any evidence of malignancy or squamous intraepithelial lesions. However, a DNA test for HPV was positive. Genotype-specific testing was not performed, but clinically, the lesions appeared non-dysplastic and consistent with low-risk HPV. The patient had not received the HPV vaccination before presentation, which may have increased her susceptibility.

Given the manifestation of the cutaneous lesions, she was referred to a dermatologist, who diagnosed seborrheic keratosis, nevi, and papillomatous lesions, likely associated with immunomodulation due to fingolimod therapy. The diagnosis of seborrheic keratosis was made clinically, based on characteristic features.

After careful consideration, the decision was made to discontinue fingolimod due to its association with increased susceptibility to HPV and other viral skin infections. As an alternative, the patient was given dimethyl fumarate. The patient was also referred for dermatologic follow-up and HPV monitoring. All the lesions resolved completely within two months of discontinuing fingolimod.

## Discussion

Fingolimod is a structural analog of natural sphingosine. It undergoes phosphorylation in vivo to produce fingolimod-phosphate, which binds to the S1PR. These receptors play a crucial role in regulating lymphocyte trafficking by retaining lymphocytes in lymphoid tissues and recirculating them back into the blood. Fingolimod-phosphate causes internalization and degradation of S1PRs, leading to a reduced receptor density. This downregulation renders lymphocytes unresponsive to the physiological S1P gradient, therefore depriving them of the essential signal required to exit lymphoid tissues. As a result, lymphocytes become sequestered within the lymph node, leading to peripheral lymphopenia and limiting their ability to infiltrate the CNS [3].

Fingolimod primarily affects T cells and prevents the egress of naïve T cells and central memory T cells from the lymph node, and B cell trafficking as well - both of which are believed to be important for inducing neurological damage in MS patients [3,8]. These immunological changes make patients more susceptible to specific viral infections [9]. Notably, fingolimod does not significantly affect effector T cells, nor does it impair T cell activation, proliferation, differentiation, or cytokine production. As a result, immune responses governed by these mechanisms remain largely intact [3,8]. The AEs associated with fingolimod stem from the immunomodulatory effects and should therefore be anticipated in systems regulated by these affected pathways [8]. Dermatologic manifestations are among the reported AEs.

In our case, cutaneous cytology helped exclude malignancy or squamous intraepithelial lesions. A DNA test for HPV was positive. The onset of cutaneous manifestations four months after initiating fingolimod therapy, along with the exclusion of other potential etiologies, supported the likelihood that these dermatologic findings were an AE of fingolimod. Furthermore, following discontinuation of the medication, the patient's skin lesions resolved completely within two months.

Given the temporal association between fingolimod initiation and the onset of cutaneous lesions, we assessed causality using the Naranjo Adverse Drug Reaction Probability Scale [10]. The patient scored 6, indicating a "probable" adverse reaction. This was based on the timing of symptom onset, improvement after discontinuation, lack of alternative explanations, and supporting literature.

A literature search yielded a case report of a patient who developed melanoma 61 months after initiating fingolimod, while another documented melanoma onset just two months after starting the medication [6,11]. Triplett et al. (2019) described five patients who developed various HPV-related warts, with the onset ranging from 17 to 58 months after treatment initiation [4]. Mhanna et al. (2021) reported 16 patients with HPV-associated lesions, with a mean onset time of 4.1 years (SD = 2.7, range: 0.2-12 years) [7].

Additionally, Brusco et al. (2018) described two cases of rosacea that developed shortly after beginning treatment [5]. Considering the findings mentioned above, the development of papillomas caused by HPV infection is not surprising, as 2.2% of patients treated with fingolimod develop HPV infection [12]. However, the abrupt onset of multiple seborrheic keratoses was unexpected. Similarly, the development of warts within four months of initiating fingolimod therapy was less typical and underlines the importance of dermatologic monitoring in fingolimod-treated patients.

## Conclusions

This case highlights the importance of dermatologic monitoring in patients receiving fingolimod, as skin manifestations may arise due to its immunomodulatory effects. HPV-related papillomas are recognized dermatological AEs of fingolimod and can appear within a few months of treatment initiation. In our case, the patient developed a rare dermatologic manifestation of fingolimod - seborrheic keratosis. This emphasizes the importance of identifying and managing dermatologic complications in MS patients taking fingolimod. Regular skin evaluations are essential to ensure timely intervention and appropriate adjustments in therapy when necessary.
